# Combined Replacement of Aortic Valve and Ascending Aorta—A 70-Year Evolution of Surgical Techniques

**DOI:** 10.1055/s-0041-1729913

**Published:** 2021-10-11

**Authors:** Igor Vendramin, Uberto Bortolotti, Davide Nunzio De Manna, Andrea Lechiancole, Sandro Sponga, Ugolino Livi

**Affiliations:** 1 Division of Cardiac Surgery, Cardiothoracic Department, University Hospital, Udine, Italy

**Keywords:** aorta, ascending aorta, ascending aorta replacement, aortic valve replacement

## Abstract

Simultaneous replacement of the ascending aorta and aortic valve has always been a challenging procedure. Introduction of composite conduits, through various ingenious procedures and their modifications, has changed the outlook of patients with aortic valve disease and ascending aorta pathology. In the past 70 years, progress of surgical techniques and prosthetic materials has allowed such patients to undergo radical procedures providing excellent early and long-term results in both young and elderly patients. This article aims to review the most important technical advances in the treatment of aortic valve disease and ascending aorta aneurysms recognizing the important contributions in this field.

*“There is no disease more conducive*

*to clinical humility than aneurysm of the aorta.”*

Sir William Osler

## Introduction

In patients with aortic valve disease, dilatation of the ascending aorta is frequently present. Severe calcific aortic stenosis may cause aneurysm of the ascending aorta as a consequence of poststenotic dilatation; moreover, aortic insufficiency is commonly associated with aortic root and/or ascending aorta enlargement in patients with genetically inherited collagen tissue disorders. 1 At present, simultaneous treatment of aortic valve disease and ascending aortic aneurysms has become commonplace, either if the lesions are amenable to repair or indicate replacement. 2 3 However, many years ago, these patients could represent a challenging problem, and to reach the current low risks and excellent results of such combined procedures, a long pathway, full of attempts, failures, and eventual successes, has been traced. This exciting journey is retraced in the following review.

## Historical Background

Until early 1950, treatment of ascending aorta aneurysms, a pathology already known to the physicians of ancient Egypt, consisted mainly in efforts to strengthen or reinforce the aortic wall to retard its growth or to promote aneurysm thrombosis by introducing foreign materials. 4 In 1952, Cooley and De Bakey 5 reported a small series of patients with intrathoracic aneurysms of the aorta and great vessels, treated with various techniques including wrapping, ligation, and aneurysmorrhaphy. In 1956, Cooley and De Bakey, 6 reported the first case of a fusiform aneurysm of the ascending aorta resected and replaced with an aortic homograft using cardiopulmonary bypass. This operation was made possible by the recent introduction of the heart–lung machine, while a homograft was used due to the unavailability of fabric grafts. Later, Bahnson and Nelson 7 and McKusik et al 8 were among the first to describe the association of cystic medial necrosis with aortic aneurysms, amenable to surgical repair, aortic valve disease, and aortic dissection. Interestingly, the association between aortic valve regurgitation and aortic root aneurysm as the cause of valvular malfunction had been already observed by Corrigan, 9 some 25 years earlier.

## Subsequent Developments

The middle-to-late 1950s witnessed the sunrise of the modern aortic surgery. Surprisingly, some intrathoracic aortic pathologies, such as ascending aorta or arch aneurysms, were repaired much earlier than cardiac valve diseases. In the early 1920s, Cutler in Boston and Souttar in London, with their first pioneering attempts to palliate mitral valve stenosis, stimulated an increasing interest in valvular surgery. In 1952, Hufnagel and Harvey 10 had implanted the first ball–valve prosthesis in the descending thoracic aorta in a patient with aortic insufficiency through a left thoracotomy. After the heart and lung machine became available, in 1960, Harken et al 11 reported the first successful replacement of the aortic valve implanting a caged-ball mechanical prosthesis in the subcoronary position. 11 The Harken–Soroff prosthesis was made by two concentric stainless steel cages to avoid impingement on the aortic wall, containing a silicone rubber ball; an Ivalon patch attached to the ring was used to close the aortotomy incision. This milestone operation started the history of prosthetic cardiac valve substitutes.

## Combining the Two Procedures

### The Wheat Technique

**Fig. 1:**
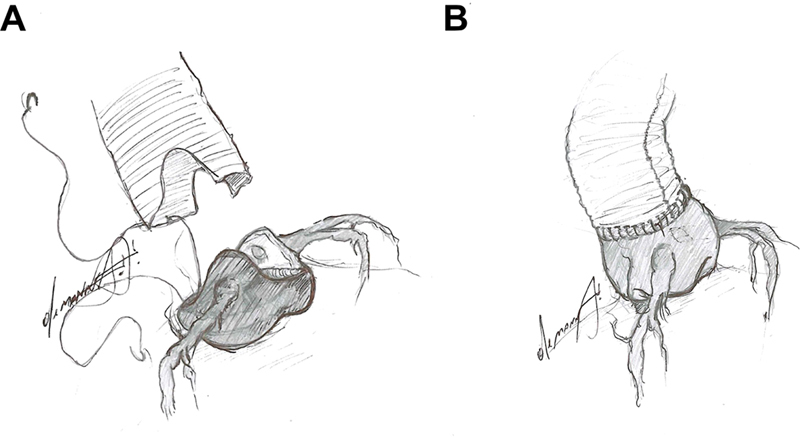
( **A** ) Schematic drawing showing preparation of the aortic root for the classical “Wheat procedure.” ( **B** ) Supracommissural replacement of the ascending aorta, currently considered as the “Wheat procedure.”

The need to treat patients presenting with combined aortic valve pathology and aneurysms of the ascending aorta by a single operation soon became apparent. A solution to this problem was first proposed by Wheat et al in 1964 who described a technique of aortic valve replacement (AVR) associated with supracoronary replacement of the ascending aorta. In a patient with syphilitic aortic aneurysm “the aortic aneurysm was excised entirely down to the aortic annulus except for two tongues of the aortic wall which contained the coronary ostia.” The aortic valve cusps were then removed and a Starr–Edwards caged-ball prosthesis implanted. “A woven Teflon graft was fashioned to accommodate the aortic wall tongues containing the coronary artery orifices. The graft was attached to the sewing ring of the previously seated prosthetic valve and the cut edge of the aorta.” 12 Despite this quite clear description ( Fig. 1A ), the supracoronary Wheat technique has often been misinterpreted and even recently confused with what is currently erroneously considered as true supracoronary when it should be more properly named supracommissural 13 14 ( Fig. 1B ). Indeed, in the original Wheat technique, all aortic sinuses were excised while in the less radical supracommissural procedure, the sinuses are left in place. A supracommissural approach was certainly that presented by Groves et al 15 at the 44th Annual Meeting of the American Association for Thoracic Surgery in 1964 reporting the surgical management of aortic insufficiency secondary to aneurysms of the ascending aorta. Interestingly, in discussing that paper, Wheat et al 12 stressed the need for total excision of the aortic root when the aneurysm extended to the proximal subcoronary portion and to reattach the coronary buttons. Therefore, all those who present in their reports data on supracoronary replacement of the ascending aorta do not perform this operation as described by Wheat et al, 12 at least according to his original drawings, but rather a simpler supracommissural replacement. Probably, this is just a matter of mere etymology, but these two similar operations could entail different technical difficulties and certainly a more radical approach to a specific aortic disease. In fact, the rationale for a supracoronary Wheat procedure was not to leave diseased aortic tissue in place which could subsequently dilate requiring reoperation.

For such reason it is difficult to obtain adequate information on the results of the original technique apart from that provided by the author himself. In a series of 13 patients, there was a hospital mortality of 15% (which was considered reasonable), while seven died during 1 month to 5 years after surgery for unspecified causes. Most likely because the Wheat operation was later replaced by other procedures, long-term follow-up data are lacking.

### The Bentall–De Bono Technique

Combined AVR and ascending aorta using a composite conduit was popularized by Bentall and De Bono 16 in 1968, when a new technique, which subsequently would have been known as (only) the Bentall procedure, was devised. In this technique, the aneurysm was opened and the aortic valve excised. A Starr–Edwards prosthesis was sewn at the end of a Teflon graft and implanted in the aortic annulus. The coronary ostia were then reattached to the graft since “the aortic wall was sutured to the perimeter of the holes in the Teflon tube, thus reincorporating the coronary ostia within the new aorta.” Finally, “the wall of the aneurysm was closed over the prosthesis” for hemostatic purposes. Two years later, Edwards and Kerr 17 described a technique almost identical to the Bentall procedure without giving credit to the previously published work; they considered their procedure “safer,” probably compared with the Wheat operation since this was the only reference quoted. A sort of the hemi-Wheat procedure was described in 1973 by Najafi 18 in two patients with aneurysm of the ascending aorta and severe displacement of the right coronary ostium; after insertion of a tilting-disc aortic prosthesis, a graft was sutured to the remnant of the aorta and trimmed at the left coronary ostium level while the right ostium was attached to the graft by interposition of a segment of saphenous vein.

The Bentall procedure increased the surgical complexity, and postoperative bleeding was recognized as a problem in an operation with so many suture lines. Maintaining the aneurysmal aortic wall in place and wrapping it tightly around the aortic graft could facilitate hemostasis but in case of perigraft bleeding, tension on the sutures could favor false aneurysm formation mainly at the coronary anastomosis. 19 For such reason, a modification of the classic Bentall procedure was proposed where the coronary ostia were detached with the surrounding aortic tissue, widely mobilized, and individually sutured to the aortic graft. 20 This modification, known as the “button Bentall” or “modified Bentall” procedure (MBP), consistently reduced pseudoaneurysm formation, minimizing the risk of coronary artery kinking.

Through the years, further modifications were introduced mainly to deal with the problem of severely displaced coronary arteries. These included insertion of a composite conduit and saphenous grafts to connect one or both coronary ostia to the graft, interposition of a segment of a small vascular graft as approach to a short-left coronary artery, or standard coronary artery bypass grafting after occlusion by sutures of the coronary ostia. 21 22

**Fig. 2:**
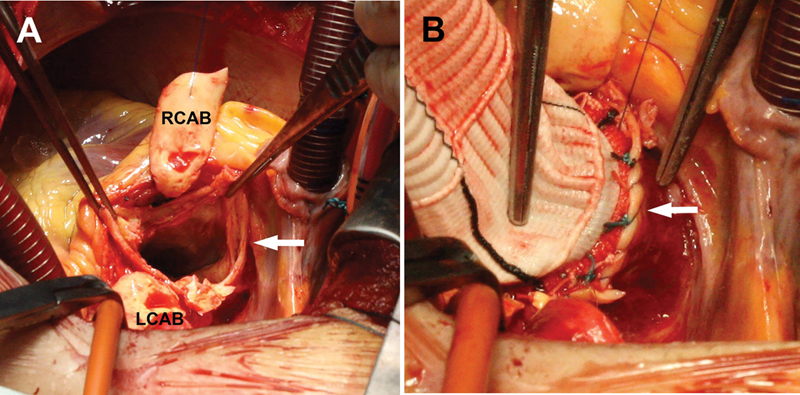
( **A** ) Preparation for the modified Bentall procedure with excision of the aortic valve cusps and ascending aorta. Wide left coronary artery button (LCAB) and right coronary artery button (RCAB) are isolated and a rim of the supravalvular aortic wall is left (arrow). ( **B** ) After the conduit has been fixed, a running polypropylene suture joins the prosthetic sewing ring and the aortic rim, and is completed circumferentially (arrow).

**Fig. 3:**
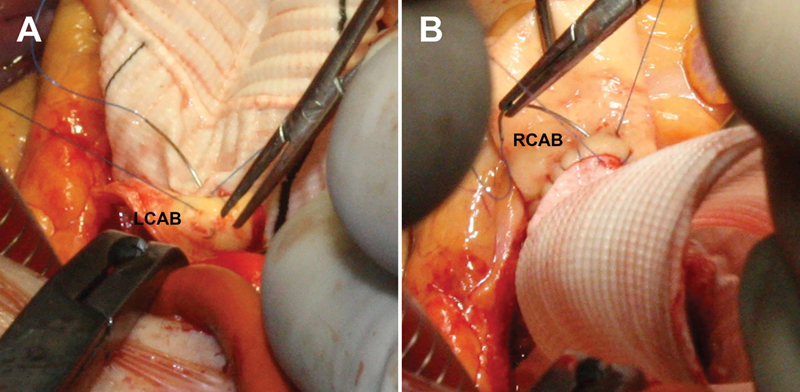
The wide coronary buttons are reattached to the graft with a double layer running suture, starting with the left coronary artery button (LCAB) ( **A** ); ( **B** ) the right coronary artery button (RCAB) is then reattached using the same technique.

**Fig. 4:**
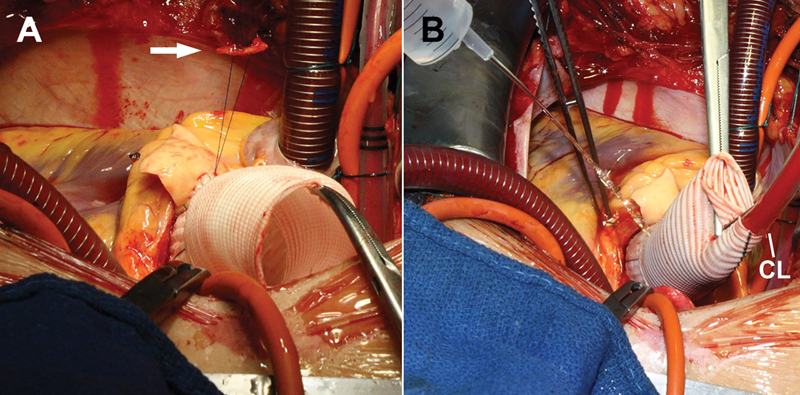
( **A** ) At the end, the suture is knotted over a small pledget of autologous pericardium (arrow). ( **B** ) Prior to the distal anastomosis the graft is pressurized through the cardioplegia line (CL) to verify hemostasis.

To reduce the issue of postoperative bleeding after the MBP, a large variety of technical tips and tricks have been proposed. Bleeding at the proximal graft anastomosis can be controlled by reinforcing the suture with a strip of Teflon or pericardium or modifying the conduit by the miniskirt or the flanged techniques. 23 24 25 26 Extremely effective has proved the use of an additional suture to reinforce the proximal suture line. 27 28 Once the aortic valve is excised, the aneurysm is removed leaving 3 to 5 mm of the periannular aortic wall ( Fig. 2A ); after the conduit has been fixed to the aortic annulus, a continuous suture of 3/0 polypropylene is used to join the prosthetic ring and the remnant of the aortic wall ( Fig. 2B ). To avoid bleeding at the coronary ostia anastomosis, sutures can be reinforced with autologous pericardium or Teflon donuts, especially in the presence of fragile tissue such as in aortic dissection or Marfan's patients. 29 In most cases, however, additional material is not necessary; if the coronary buttons are harvested with as much periaortic tissue as possible, the coronary anastomosis to the graft can be performed in a double-layer fashion to achieve enough strength to prevent leakages 28 ( Figs. 3 and 4 ). At the end of the procedure, prior to performing the distal suture, the aortic root can be pressurized using the cardioplegia line to detect any proximal significant bleeding source which can be easily corrected at this time, since after release of the aortic cross-clamp, certain bleeding sites would be almost inaccessible 30 ( Fig. 4 ). The MBP, as most of the operations involving the ascending aorta, is generally performed through a standard median sternotomy. However, particularly skilled surgeons may perform it through a minimally invasive approach; in such cases, the term “mini-Bentall” has been coined.

The MBP has demonstrated excellent results even at extended follow-up. 2 Conduits with a mechanical prosthesis have been generally used showing that the MBP provides a stable correction and significant freedom from valve-related complications. 2 31 Currently biological conduits are more frequently used due to the increasingly number of elderly patients requiring AVR and ascending aorta replacement. In elderly patients, biological conduits are particularly indicated for an MBP due to avoidance of anticoagulation and since they are likely to outlive an old recipient. In younger patients, mechanical and biological conduits provide substantially similar results, making the choice difficult in the absence of specific guidelines. 32

### The Cabrol Technique

As alternative to the MBP, Cabrol et al, 33 in 1981, proposed a new surgical approach to replace the ascending aorta with reimplantation of the coronary arteries which was applied in an initial series of 30 patients. After implantation of a valved conduit in the aortic annulus, the coronary ostia were connected to the neoaorta by a 8mm Dacron graft. “The connection to the left coronary ostium is established initially and the Dacron tube is led circumferentially around the right flank of the aortic prosthesis anteriorly to where it is anastomosed to the right coronary ostium.” At the end of the procedure, the aortic wall was wrapped around the graft creating a fistula between the right atrium and the aneurysmal sac to drain any blood accumulation. Although this technique could, in some cases, facilitate tension-free coronary anastomosis to the graft, one major drawback was that the entire coronary flow depended on a single source, with potentially catastrophic consequences in case of graft thrombosis. 34 Therefore, also the Cabrol technique underwent subsequent modifications. Piehler and Pluth, 35 almost simultaneously to the publication of Cabrol's paper, performed probably the first hemi-Cabrol procedure connecting the right coronary ostium directly to the graft and the left coronary ostium through a short graft of Gore-Tex. Fifteen years later, Mills et al 36 modified the Cabrol procedure with the “legs” technique by direct implantation of short separate grafts from each coronary artery ostium into the composite aortic graft. Other modifications included anastomosis of the graft to the right coronary artery directly to the valved conduit, and the graft to the left main to the previous aortocoronary graft in a T-fashion or the Cabrol-like anastomosis of the radial artery to both the right coronary and obtuse marginal arteries. 37 38

Early and long-term results of the Cabrol technique appear to be inferior to those observed with the MBP. One of the longest follow-up evaluations was provided by Gelsomino et al 39 who reported a 10-year survival of 59% and not negligible incidence of early and long-term complications. Therefore, the Cabrol technique is still considered as an important procedure only in selected cases, mainly in complex aortic disease. 34 This has been demonstrated by its successful use in patients with a porcelain aorta 40 and in complex reoperations. 41

## Conclusion

From this review, it appears evident how the use of composite conduits for AVR and replacement of the ascending aorta in a single operation, especially with the MBP, has represented for many years the gold-standard treatment for patients with aortic valve disease and aneurysms of the aortic root. The use of biological conduits for MBP is steadily increasing due to the continuous aging of patients referred for surgery. Novel procedures aimed to replace the aortic root preserving the aortic valve in case of aortic insufficiency are currently widely used but whenever the aortic valve is considered not amenable of repair, the MBP remains the procedure of choice. Furthermore, the recent introduction of the Valsalva graft, both in composite conduits manufacture and in valve-sparing root replacement operations, has significantly facilitated coronary ostia reimplantation into the graft almost eliminating the need for associated cumbersome procedures. In addition, since in patients with calcific aortic stenosis and ascending aorta dilatation, as well as in those with pure aortic insufficiency, transcatheter procedures are at present still contraindicated, surgical repair remains the only possible option in this setting. Finally, this review has also provided the opportunity to clarify and specify some technical details and to give credit to those who made important contributions on this issue.
